# Molecular PET/CT Profiling of ACE2 Expression In Vivo: Implications for Infection and Outcome from SARS‐CoV‐2

**DOI:** 10.1002/advs.202100965

**Published:** 2021-06-26

**Authors:** Hua Zhu, Hanwen Zhang, Nina Zhou, Jin Ding, Jinquan Jiang, Teli Liu, Ziyu Liu, Feng Wang, Qian Zhang, Zhuochen Zhang, Shi Yan, Lei Li, Nadia Benabdallah, Hongjun Jin, Zhaofei Liu, Lisheng Cai, Daniel L. J. Thorek, Xing Yang, Zhi Yang

**Affiliations:** ^1^ Key Laboratory of Carcinogenesis and Translational Research, NMPA Key Laboratory for Research Evaluation of Radiopharmaceuticals (Ministry of Education/Beijing) Department of Nuclear Medicine Peking University Cancer Hospital and Institute Beijing 100142 China; ^2^ Department of Radiology Washington University in St. Louis School of Medicine St. Louis MO 63110 USA; ^3^ Program in Quantitative Molecular Therapeutics Washington University in St. Louis School of Medicine St. Louis MO 63110 USA; ^4^ Department of Nuclear Medicine The Affiliated Hospital of Inner Mongolia Medical University Hohhot 010050 China; ^5^ Department of Nuclear Medicine Peking University First Hospital Beijing 100034 China; ^6^ Key Laboratory of Carcinogenesis and Translational Research (Ministry of Education/Beijing) Department of Thoracic Surgery II Peking University Cancer Hospital and Institute Beijing 100142 China; ^7^ Beijing Tongren Eye Center Beijing Tongren Hospital Capital Medical University Beijing Ophthalmology and Visual Scientific Key Lab Beijing 100730 China; ^8^ Guangdong Provincial Key Laboratory of Biomedical Imaging Fifth Affiliated Hospital Sun Yat‐sen University Zhuhai Guangdong Province 519000 China; ^9^ Medical Isotopes Research Center and Department of Radiation Medicine School of Basic Medical Sciences Peking University Health Science Center Beijing 100191 China; ^10^ Molecular Imaging Branch National Institute of Mental Health National Institutes of Health Bethesda MD 20892 USA; ^11^ Department of Biomedical Engineering Washington University in St. Louis St. Louis MO 63110 USA

**Keywords:** angiotensin‐converting enzyme 2, human, positron emission tomography imaging, severe acute respiratory syndrome coronavirus‐2, translational research

## Abstract

Rapid progress has been made to identify and study the causative agent leading to coronavirus disease 2019 (COVID‐19) but many questions including who is most susceptible and what determines severity remain unanswered. Angiotensin‐converting enzyme 2 (ACE2) is a key factor in the infection process of severe acute respiratory syndrome coronavirus‐2 (SARS‐CoV‐2). In this study, molecularly specific positron emission tomography imaging agents for targeting ACE2 are first developed, and these novel agents are evaluated in vitro, in preclinical model systems, and in a first‐in‐human translational ACE2 imaging of healthy volunteers and a SARS‐CoV‐2 recovered patient (NCT04422457). ACE2 expression levels in different organs in live subjects are quantitatively delineated and observable differences are measured in the patient recovered from COVID‐19. Surprising sites of uptake in the breast, reproductive system and very low uptake in pulmonary tissues are reported. This novel method can add a unique tool to facilitate SARS‐CoV‐2 related research and improve understanding of this enigmatic disease. Molecular imaging provides quantitative annotation of ACE2, the SARS‐CoV‐2 entry receptor, to noninvasively monitor organs impacted by the COVID‐19.

## Graphical abstract

Severe acute respiratory syndrome coronavirus‐2 gains infect cells through binding to the angiotensin‐converting enzyme 2 (ACE2) receptor. Using a high affinity radiolabeled peptide to the receptor, the whole body and quantitative determination of ACE2 expression can be imaged. This molecular imaging approach enables animal model and clinical evaluation of this critical receptor, noninvasively.

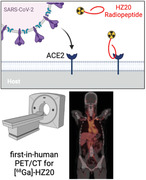

## 1 Introduction

The ongoing epidemic caused by severe acute respiratory syndrome CoV‐2 (SARS‐CoV‐2) has emerged as a global health and economic crisis of unprecedented magnitude.^[^ ^1^ ^]^ The World Health Organization (WHO) officially declared the novel coronavirus disease 2019 (COVID‐19) a pandemic on March 11, 2020.^[^ ^2^ ^]^ To date, more than 106.2 million cases and over 2.31 million deaths have been attributed to COVID‐19 worldwide.^[^ ^3^ ^]^ The majority of the infected exhibit mild or no symptoms. However, multiple organs are at risk of rapid and sustained damage and failure in those who have adverse manifestations of the disease. Dozens of studies, many ongoing, have attempted to clarify the susceptibility of COVID‐19 and the risk factors contributing to its serious complications.^[^ ^4^, ^5^ ^]^ While age and several co‐morbidities have been identified, the basis of this variability in severity is unknown and effective cure for the infection remains in urgent development.^[^ ^6^, ^7^, ^8^ ^]^

The spike glycoprotein of SARS‐CoV‐2 plays the critical role in mediating viral entry into host cells after binding to the human angiotensin‐converting enzyme 2 (ACE2).^[^ ^9^, ^10^, ^11^ ^]^ Since SARS‐CoV‐2 must bind with ACE2 before entering the host cells in humans, the distribution and expression of ACE2 is critical for SARS‐CoV‐2 infecting these target tissues. Accumulating evidence has demonstrated the implication of ACE2 in the pathological progression in tissue injury and several chronic diseases.^[^ ^12^, ^13^, ^14^, ^15^ ^]^ The quantification of ACE2 distribution and expression level is of importance for the risk profile of COVID‐19—may play a role in monitoring therapies—with potential value in monitoring of the recovered, many of whom display long‐term sequelae including muscle weakness, cognitive, and respiratory issues.^[^ ^59^, ^60^, ^61^, ^62^ ^]^

SARS‐CoV‐2 infection is primarily thought to initially infect the respiratory tract, supported by ACE2 expression in nasal and lung alveolar epithelial cells.^[^ ^16^, ^17^ ^]^ Medium to high expression of ACE2 has also been reported in kidney, heart muscle, intestine, and reproductive system, which have also been identified as sites of complications from SARS‐CoV‐2 infection.^[^ ^16^, ^18^ ^]^ However, existing methods like immunohistochemistry (IHC), RT‐PCR, and RNA‐seq can only provide analysis of samples ex vivo, which limits their clinical applications in live subjects especially when temporal resolution is required for monitoring the progression of the disease.^[^ ^16^, ^17^, ^18^, ^19^ ^]^

**Figure 1:**
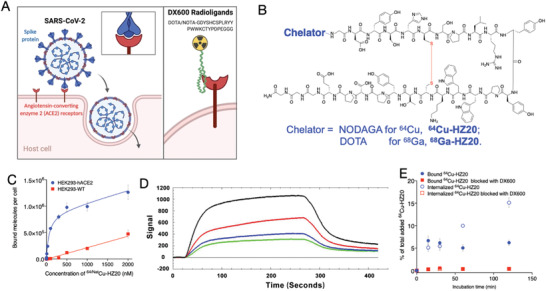
In vitro characterization of ACE2 targeting radioligands. A) DX600 and radiolabeled DX600 analogs may interface the penetration of SARS‐CoV‐2 into host cells by specifically binding to ACE2 receptor. B) The structure ACE2 targeting peptides. C) Saturation binding assay of ^64/Nat^Cu‐HZ20 over HEK293‐hACE2 and HEK293‐WT (non‐transduced) at 4°C. D) Binding assay of HZ20. E) Time‐dependent binding and internalization of ^64^Cu‐HZ20 over HEK293‐hACE2 cells at 37 °C.

Molecular imaging has emerged as a non‐invasive approach to quantitatively monitor biochemical processes occurring inside organisms in real time. Positron emission tomography (PET) utilizes radioligands to interrogate disease‐relevant biomarkers or pathways providing quantitative spatial distribution of physiologically relevant targets with exquisite sensitivity.^[^ ^20^, ^21^ ^]^ With the long‐term aim to determine the effects of ACE2 expression on SARS‐CoV‐2 infection and COVID‐19 severity, we initiated this study to develop ^64^Cu and ^68^Ga‐labeled peptides to specifically targeting ACE2. We have developed a class of ACE2‐specific PET radioligands with excellent imaging and pharmacokinetic properties. Preclinical model systems were used to validate and optimize this approach to quantify ACE2 expression in vivo using ^68^Ga‐ and ^64^Cu‐labeled analogs of the ACE2‐targeting DX600 peptide:, ^68^Ga‐HZ20 and ^64^Cu‐HZ20, respectively (**Figure** **1B**). The human‐specific ACE2 inhibiting cyclic peptide DX600 was ^68^Ga‐HZ20 that was advanced to the clinic for PET/CT imaging and organ‐based standardized uptake value (SUV analysis) of 20 healthy volunteers. A patient who had recovered from SARS‐CoV‐2 infection was included, and demonstrated greater ^68^Ga‐HZ20 SUV_max_ (a standardized measure of tracer accumulation). Complementing pathological analyses, these intriguing results are expected to demonstrate that radiolabeled HZ20 analogs have substantial value in quantifying the ACE2 distribution in the entire body, and monitoring the transient upregulation of ACE2 expression to guide patient management and evaluate therapeutic intervention of COVID‐19 patients.

## 2 Results

DX600 is a high affinity ACE2 binding cyclic peptide with an intramolecular disulfide bond initially discovered from a phage display screen for ACE2 inhibition.^[^ ^22^ ^]^ It shows high selectivity on ACE2 over ACE and is stable to ACE2 catalyzed hydrolysis. We hypothesized the potency of the ligand would not be significantly diminished through modification with a radio‐metal chelator, such as 1‐(1,3‐carboxypropyl)‐1,4,7‐ triazacyclononane‐4,7‐diacetic acid (NODAGA) for stable ^64^Cu labeling or 1,4,7,10‐tetraazacyclododecane‐1,4,7,10‐tetraacetic acid (DOTA) for stable ^68^Ga labeling, at the N‐terminus of DX600 as it is distant from the key binding domain of CSPLRYYPWWKC. Chelate conjugated‐DX600 was obtained in high purity (>98%) and characterized by mass spectrometry (Figure 1 and Figure S1, Supporting Information), which are matching with the structure‐based estimation. ^64^Cu‐HZ20 (Figure 1B) was prepared quantitatively with a radiochemical purity of >97% within 30 min. ^68^Ga‐HZ20 (Figure 1B) was produced in high radiochemical yield of 59.9 ± 3.9% (non‐decay corrected, *n* = 10) with over 95% radiochemical purity. The specific activity of ^68^Ga‐HZ20 was determined to be 6.0 × 10^4^ GBq mmol^−1^ (*n* = 3). ^64^Cu/^68^Ga‐HZ20 with different decay half‐lives (12.7 h vs 68 min) allowed us to conveniently carry out the further studies.

We initially tested the binding potency by using surface plasmon resonance (SPR). DX600 and HZ20 showed similar *K*_d_ of 98.7 and 100.0 nm, respectively (Figure 1D and Figure S2B, Supporting Information). The specificity of binding was assessed using a saturation binding assay with ^64/Nat^Cu‐HZ20 on ACE2‐expressing and vehicle HEK293 cells at 4 and 37 °C, respectively. ^64/Nat^Cu‐HZ20 displayed a higher binding potency toward ACE2 (66 ± 1 nm) at 4 °C than the parental DX600 (Figure 1C), a lower affinity (143 ± 1 nm) at 37 °C with higher binding molecules (Figure S2A, Supporting Information), which indicated that the internalization of ^64^Cu‐HZ20 might exist at physiological conditions, and may be retained in target cells. Our further in vitro cell uptake studies showed that the radiolabeled ACE2‐binding peptide displayed a highly specific accumulation ACE2‐expressing cells in a time dependent manner with significant retention (Figure S2A, Supporting Information), and 71 ± 4% of total bound ^64^Cu‐HZ20 by was internalized (Figure 1E).

**Figure 2:**
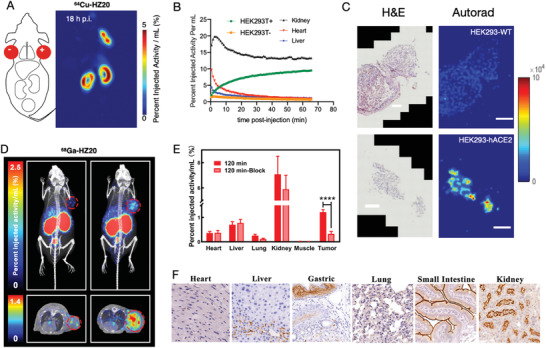
In vivo PET imaging of hACE2‐positive pseudotumors with ^64^Cu‐HZ20 and ^68^Ga‐HZ20 and its ex vivo assays. A) Dual xenografts (left: HEK293‐WT; right: HEK293‐hACE2) imaged with ^64^Cu‐HZ20 at 18 h post injection. B) Quantitative analysis of dynamic ^64^Cu‐HZ20 imaging of the dual xenografts, kidneys, heart and liver. C) Ex vivo autoradiographic imaging of collected dual xenografts and H&E staining after PET imaging at 18 h post injection. D) Micro‐PET/CT imaging of ^68^Ga‐HZ20 in ACE2 expressing HepG2 tumor‐bearing mice at 2 h post‐injection. The images were shown on the right, in comparison with the blocking control on the left. In the blocking group, ^68^Ga‐HZ20 was co‐injected with 50 mg kg^−1^ of DX600. The upper and lower images are MIP images and cross‐sectional images of the mice, respectively. E) Comparison of the SUV_max_ of micro‐PET imaging at 2 h post‐injection. Heart, liver, lung, kidney, muscle, and tumor were selected for both group (*n* = 4) and statistical significance could be observed for the tumor in comparison to the control group, 0.099 ± 0.0065 versus 0.024 ± 0.0041 (****p < 0.0001). F) Immunohistochemistry results of representative mouse tissue.

To evaluate the distribution, kinetics and targeted uptake of HZ20 PET at human (h)‐ACE2 sites in vivo, we evaluated two xenograft models of hACE2‐expressing cell lines, an engineered HEK293T and the liver hepatocellular carcinoma HepG2, with high endogenous ACE2 expression.^[^ ^18^ ^]^ Both radioligands enabled clear visualization of ACE2‐expressing xenografts following tail vein administration, and in vivo specificity was tested with cold blocking and ACE2‐models and non‐hACE2 expressing xenografts (**Figure** **2** and Figure S2, Supporting Information), which were also further verified by ex vivo autoradiography (Figure 2C). The percent injected activity per mL (%IA mL^−1^) of HZ20 accumulation in heart, liver, lung, kidney, muscle, and tumor at 1, 2, and 3 h were quantified by using ^68^Ga‐HZ20 PET imaging (Figure S6, Supporting Information). HepG2 tumor showed 0.56%IA mL^−1^ at 2 h, which was 96 times higher than that of muscle, and significantly blocked with co‐injection of DX600. Similar to ^68^Ga‐HZ20, ^64^Cu‐HZ20 also showed the highest signal in the kidneys, high intensity in HEK293‐hACE2 xenografts and moderate in liver (Figure 2A,B). Kinetic analyses of this longer‐lived tracer show persistent renal signal which coupled ACE2 immunohistochemistry suggest binding to the highly expressed murine ACE2 in this organ, for which radio‐HZ20 ligands have greatly reduced affinity (Figure S2, Supporting Information). We further investigated ^64^Cu‐HZ20 using hACE2 transgenic mice. Compared to the distribution of ^64^Cu‐HZ20 in the wild‐type strain, hACE2 transgenic mice showed significantly higher accumulation in the heart, lung, liver, and intestine, (Figure S3, Supporting Information) which is consistent with the tissue profile of transgenic hACE2 distribution.^[^ ^23^ ^]^

In vitro SPR and cell data and in vivo experiments clearly demonstrated the feasibility for ^64^Cu/^68^Ga‐HZ20 to specifically monitor human ACE2 expression with high contrast ratios. The capability to perform later post‐clearance imaging with cyclotron‐produced ^64^Cu (half‐life 12.7 h) and logistical advantages are offset by the increased absorbed dose computed to the kidneys. The short‐lived ^68^Ga (half‐life of 68 min) can be labeled into the DOTA chelator and is easily accessible through a network of widely available ^68^Ge/^68^Ga generators.^[^ ^24^ ^]^ In this study, we performed cGMP production of ^68^Ga‐HZ20 in the hospital, ^68^Ga‐HZ20 was proved to be stable within 2 h (Figure S1D, Supporting Information), and passed all quality control standards for clinical trial (Table S1, Supporting Information). Preclinical dosimetry was performed to determine a lead ligand for first‐in‐human evaluation, which showed that the shorter half‐life of Gallium‐68 was more amenable for human study (Figures S4–S6, Supporting Information). After an acute toxicity test proved ^68^Ga‐HZ20 to be well tolerated (Figure S5, Supporting Information), we initiated a clinical PET/CT imaging study in ten volunteers.

Five males and five females were enrolled and underwent multiple whole‐body PET/CT examinations to compute the mean organ absorbed dose per unit of administered radioactivity. The effective dose of ^68^Ga‐HZ20 to male and female were calculated as 0.017 and 0.016 mSv MBq^−1^, both of which are lower than the whole‐body effective dose of 0.019 mSv MBq^−1^ as reported for the ICRP for ^18^F‐Fluorodeoxyglucose (^18^F‐FDG, the most commonly used PET radiopharmaceutical).

**Figure 3:**
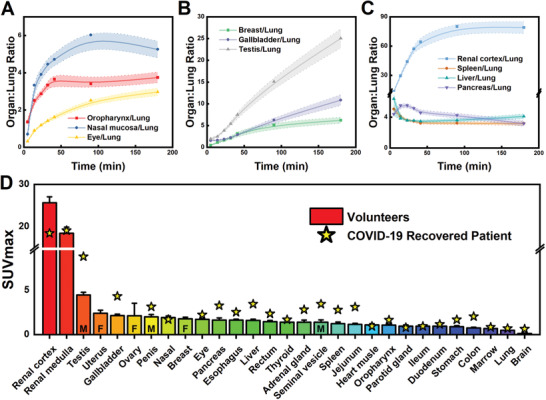
^68^Ga‐HZ20 distribution in volunteers. SUV_max_ of each organ was analyzed through the Siemens workstation (Multi‐Modality Workplace). The dynamic changes of the SUV_max_ ratio of selected‐organ‐to‐lung at seven time points (5, 14, 23, 32, 40, 90, and 180 min) were listed. A) Oropharynx, nasal mucosa, and eye, which represents the organs exposed to virus entry. B) Breast, gallbladder, and testis. C) Renal cortex, spleen, liver, and pancreas. D) Rank ordering of organ ACE2 expression in different organs indicated by SUV_max_. The average SUV_max_ from 20 healthy volunteers at 90 min scans were shown in the column (M: only for male, F: only for female). The SUV_max_ of the organs from the recovered subject at 90 min scan are indicated with a star.

With the radiation safety on ^68^Ga‐HZ20 determined, we enrolled an additional ten healthy volunteers and carried out a study in the total of 20 subjects (Table S2, Supporting Information) with the aim to quantify the ACE2 distribution in different organs. 1.85–2.96 MBq kg^−1^ of ^68^Ga‐HZ20 was applied as the injection dose. PET data were acquired for all the subjects beginning immediately after administration of ^68^Ga‐HZ20, and seven scans were performed for all the subjects (5, 14, 23, 32, 41, 90, and 180 min). The images of a representative male volunteer are shown in Figure S10, Supporting Information. The standard metric for tracer accumulation is reported at each time point, which measures the bodyweight normalized maximum concentration in a voxel within the region of interest (the standardized uptake value maximum; SUV_max_). Organs including the nasal mucosa, oropharynx, conjunctiva, heart muscle, breast, gallbladder, renal cortex, testis, seminal vesicle, corpus luteum, and digestive tract, showed both absorption and elimination phase, indicating ACE2‐specific uptake of ^68^Ga‐HZ20 (**Figure** **3A**–C and Figure S8, Supporting Information). Rapid elimination from brain, thyroid, lung, liver, spleen, adrenal gland, pancreas, and uterus, suggest relatively low ACE2 expression levels, and the radioactivity gradually clears from the blood pool (Figure 3C and Figure S8, Supporting Information). Balancing the factors of ACE2‐specific uptake and blood pool clearance we posit that static imaging at 90 min may optimally reflect the ACE2 distribution.

A static whole‐body PET/CT scan at 90 min was selected for comparative data across patients and cohorts, and the average organ SUV_max_ of ^68^Ga‐HZ20 are summarized (Figure 3D). As expected for a peptide‐based ligand, poor blood‐brain barrier penetration is noted resulting in little accumulation of activity in brain. The greatest accumulation of signal was observed in kidney (SUV_max_ of 25.67±1.39 in renal cortex and 18.34 ± 1.42 in renal medulla), which may be caused by the co‐effects of ^68^Ga‐HZ20 excretion and high kidney ACE2 expression. The reproductive system showed a relatively high SUV_max_, including uterus (2.41 ± 0.34), ovary (2.12 ± 1.42), breast (1.78 ± 0.17) for females and testis (4.46 ± 0.30), penis (2.00 ± 0.25) for males. Other organs with high SUV_max_ included nasal (1.91 ± 0.18), conjunctiva (1.71 ± 0.08), pancreas (1.63 ± 0.24), esophagus (1.63 ± 0.11), and liver (1.59 ± 0.14). There is continuing interest in the gastrointestinal system as a viral entry site, and indeed moderate radioactivity uptake was detected with the gallbladder and rectum having high SUV_max_ (2.14 ± 0.16 and 1.46 ± 0.13). However, ^68^Ga‐HZ20 PET imaging showed only low SUV_max_ in the healthy volunteer lung, even though lung is the critical organ for SARS‐CoV‐2 infection, which is consistent with the data from Human Protein Atlas database.^[^ ^18^ ^]^

**Figure 4:**
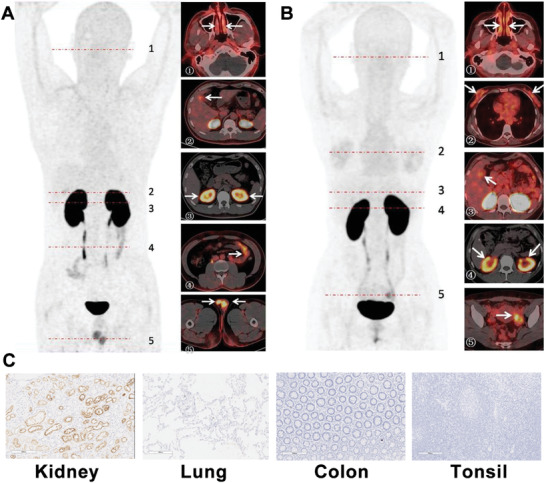
Typical PET imaging of ^68^Ga‐HZ20 in male and female volunteers. A) Radioactivity uptake in a 38‐year‐old male volunteer (# 009) at 90 min post‐injection. The MIP and transverse image showed that the nasal mucosa,^[^ ^1^ ^]^ gallbladder,^[^ ^2^ ^]^ and small intestine^[^ ^4^ ^]^ showed moderate radioactivity accumulation, and the renal cortex^[^ ^3^ ^]^ and testis^[^ ^5^ ^]^ showed high accumulation. B) Radioactivity uptake in a 34‐year‐old female volunteer (# 012) at 90 min post‐injection. The MIP and transverse image showed that the nasal mucosa,^[^ ^1^ ^]^ breast^[^ ^2^ ^]^ and gallbladder^[^ ^3^ ^]^ showed moderate accumulation, and the renal cortex^[^ ^4^ ^]^ and the corpus luteum of left ovary^[^ ^5^ ^]^ showed high accumulation. C) Immunohistochemistry analysis of ACE2 expression in normal human organs (10×).

The representative whole body PET of a 38‐year‐old male (#009) and 34‐year‐old female (#012) at 90 min post‐injection and immunohistochemical analysis of human tissues are shown in **Figure** **4**. For the male, the SUV_max_ value showed the nasal mucosa (1.57), gallbladder (0.75), and small intestine (0.48) had moderate accumulation, and the renal cortex (23.88) and testis (5.57) showed high accumulation (Figure 4A). Analysis in the female showed the nasal mucosa (2.54), breast (2.72), and gallbladder (2.76) had moderate accumulation, while the renal cortex (38.77) and the corpus luteum of left ovary (6.85) showed high accumulation (Figure 4B). The high kidney uptake is in agreement with our preclinical and human immunohistochemical findings (Figure 4C).

ACE2 distribution imaged by ^68^Ga‐HZ20 reflects sites implicated in the clinical manifestations of COVID‐19 pathology could be clearly visualized with good contrast, **Table** **1**.^[^ ^16^, ^18^ ^]^ The renal cortex, corpus luteum, and testis display high ^68^Ga‐HZ20 uptake (SUV_max_ > 2.5). The breast, gallbladder, ovary, nasal mucosa, and esophagus showed medium uptake for the young female subject (SUV_max_ 1.5–2.5), with relatively low uptake (SUV_max_ <1.5) in other organs. Additionally, the high ACE2 expression in conjunctiva as reported,^[^ ^25^, ^26^ ^]^ was also visible on PET/CT (Figure S11, Supporting Information).

**Table 1:** Comparison of ^68^Ga‐HZ20 uptake with reported ACE2 expression levels in various organs of the human body

Organs	^68^Ga‐HZ20 uptake^a)^	HPA^b)^	Expression in human tissues	Possible related clinical manifestation of COVID‐2019
Renal	High	High	High^[^ ^34^, ^35^, ^36^ ^]^	Acute kidney injuries ^[^ ^45^, ^46^ ^]^
		(renal proximal tubules)		
Testis	High	High	High^[^ ^36^ ^]^	Seminiferous tubular injuries^[^ ^47^ ^]^
		(Sertoli cells and Leydig cells)		
Corpus luteum	High	No data	No data	No data
Gallbladder	Medium	High	High^[^ ^37^ ^]^	Gastrointestinal discomfort^[^ ^45^, ^46^ ^]^
		(membranes of gallbladder epithelium)		
Intestine	Medium	High	High^[^ ^34^, ^35^ ^]^	Gastrointestinal discomfort and diarrhea^[^ ^45^, ^46^ ^]^
		(microvilli of the intestinal tract)		
Nasal mucosa	Medium	No data	High^[^ ^38^ ^]^	Olfactory deficits^[^ ^48^, ^49^ ^]^
Breast (young)	Medium	No (adipocytes, glandular cells, myoepithelial cells)	No data	Breast milk samples from nine mothers were negative (50)
Ovary	Medium	Medium (stroma cells)	High^[^ ^39^ ^]^	No data
Oropharynx	Low	No data	High^[^ ^40^ ^]^	Sore throat^[^ ^45^ ^]^
Heart muscle	Low	medium	High^[^ ^35^, ^41^ ^]^	Acute cardiac injuries^[^ ^45^, ^46^ ^]^
		(cytoplasm cardiomyocytes)		
Seminal vesicle	Low	medium	High^[^ ^42^ ^]^	No data
		(a subset of glandular cells in seminal vesicle)		
Uterus	Low	No(glandular cells, squamous epithelial cells)	Low	No data
Rectum	Low	Low	No data	Detection of viral RNA in feces^[^ ^51^ ^]^
		(glandular cells)		
Lung	Low	No	Low^[^ ^43^, ^44^ ^]^	Pneumonia^[^ ^45^, ^46^ ^]^

^a)^ High ^68^Ga‐HZ20 uptake (SUV_max_ > 2.5), medium uptake (SUV_max_ 1.5‐2.5), low uptake (SUV_max_ < 1.5);

^b)^ Data from Human Protein Atlas (HPA): cell type‐specific localization of ACE2 in human tissues based on immunohistochemistry, (https://www.proteinatlas.org/).

**Figure 5:**
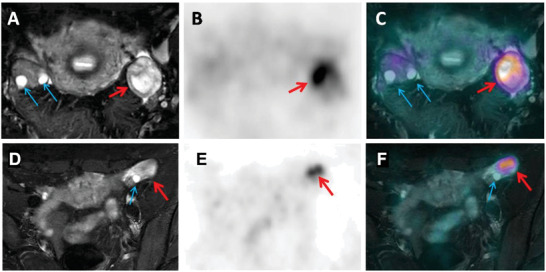
^68^Ga‐HZ20 uptake observed in luteum and confirmed with PET/MR. The scan of volunteer # 012 (A–C, top row) was taken on the 21st day of menstrual cycle and the scan of volunteer # 005 (D–F, bottom row) was taken on the 22nd day of menstrual cycle. A,D) Transvers T2WI+fs; B,E) PET; and C,F) fusion images showed both ovary foci localized in the corpus luteum (red arrow). The stroma of ovary showed slight uptake, and there was no uptake in immature follicles (blue arrow). In order to identify the precise structure of ovarian foci, PET/MR examinations were carried out at 2 h post‐injection of ^68^Ga‐HZ20 on the same day.

Interestingly, transient ACE2 uptake in the corpus luteum was observed compared to the immature follicles and ^68^Ga‐HZ20 contrast was visualized in the ovaries of two young ovulating female volunteers (#005: top row, SUV_max_ of 7.35 vs 1.95; #012: bottom row, SUV_max_ of 6.85 vs 1.93, **Figure** **5**). In order to investigate if identification of previously unidentified and transient ACE2 expression changes may be present we conducted a delayed PET/MR (2 h after ^68^Ga‐HZ20 injection) examination was carried out immediately following PET/CT imaging to reveal the precise structure of ovarian foci (Figure 5). The stroma of ovary showed slight uptake, and there was no uptake in immature follicles.

**Figure 6:**
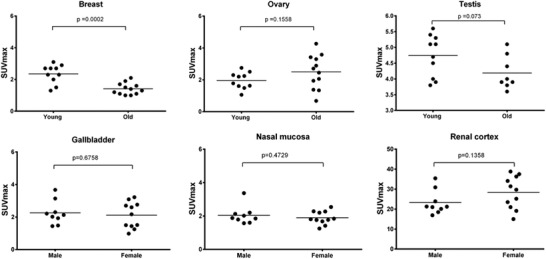
Comparison of SUV_max_ values of organs at 90 min. Top row: The comparison of reproductive system organs between young (<50 years old) and old (≥50 years old) volunteers shows that the young group has a significant higher uptake in the breast than the old, but no significant difference was observed in ovaries and testes. Bottom row: The comparison of high uptake organs (gallbladder, nasal mucosa, and renal cortex) between male and female shows no significant difference.

Clinical severity and mortality of COVID‐19 has been more severe for men than women, in several studies^[^ ^27^, ^28^ ^]^ and the aged population is particularly susceptible.^[^ ^29^, ^30^ ^]^ To investigate the relationship of ACE2 across these populations we compared organ uptake by ^68^Ga‐HZ20 PET (**Figure** **6**, Figure S12, Supporting Information). A strong correlation was discovered between the ACE2 expression in the breast of female volunteers and with age^[^ ^31^, ^32^ ^]^ (50‐year as the cutoff), with the young group higher than the old group (2.21 ± 0.25 vs 1.35 ± 0.22, *p* = 0.0002, at 90 min, Figure 6). At this time, we continue to increase the sample size to confirm these and other observations, a limitation of this first‐in‐man study.

**Figure 7:**
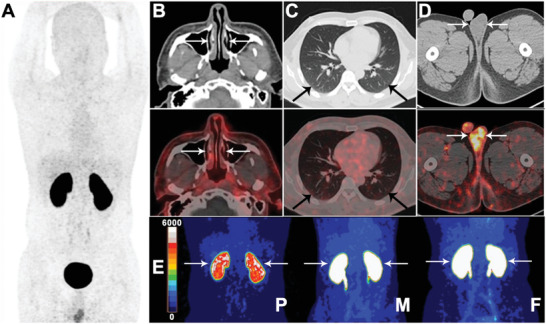
Typical PET imaging of the volunteer recovered from COVID‐19 infection. A 38‐year‐old male infected with COVID‐19 and recovered was included in the study. A) The whole body PET image and B–D) axial PET/CT images of nasal, lung, and testis at 90 min post‐injection were shown. The renal cortex showed obviously lower uptake of ^68^Ga‐HZ20 than healthy male and female volunteers (E; MIP with the same scale bar, P = Patient, M = Male, F = Female). The SUV_max_ of renal cortex were lower than healthy volunteers (SUV_max_ of 11.59 vs 22.18 ± 1.78 for the left kidney, 18.40 vs 25.67 ± 1.39 for the right kidney). The gallbladder and testis showed higher uptake than healthy volunteers (SUV_max_ of 4.32 vs 2.14 ± 0.39 for gallbladder, 8.79 vs 4.51 ± 0.54 for the right testis, 8.65 vs 4.58 ± 0.40 for the left testis), and the SUV_max_ of other organs were listed in Figure 3D compared with the healthy volunteers. The arrows indicate the nasal mucosa in (B), the lungs in (C), the testes in (D), and the kidneys in (E).

We next tested ACE2 PET in a 38‐year‐old male infected SARS‐CoV‐2 at 7 months post infection. Images at 90 min post‐injection are shown in **Figure** **7** and all organs with high radioactivity uptake were visualized clearly with SUV_max_ of each organ determined (Figure 3D). Evaluation of a larger number of recovered patients is required to draw robust conclusions, here the gallbladder, testis, and many of the normal organs showed increased uptake versus the healthy volunteer pool. Conversely, renal cortex accumulation of the tracer was substantially lower (SUV_max_: 18.40 vs 25.67 ± 1.39, see Supporting Information videos for details). Acute kidney injury has been reported in about 9% patients hospitalized with COVID‐19,^[^ ^33^ ^]^ and these results were unexpected. While speculative, the differences in this patient suggest an organ‐specific response, at the molecular expression‐level, resulting from infection even at these extended times post‐infection. Extension and verification of these results in a wider population of COVID‐19 patients will provide additional insight.

## 3 Discussion

The ACE2 expression level and organ‐specific distribution may potentially reflect the susceptibility, severity, and prognosis of SARS‐CoV‐2 infection. A non‐invasive imaging tool to explore expression characteristics of ACE2 in vivo may assist in understanding the pathogenesis of SARS‐CoV‐2 infection with the potential to assist in development of mitigation and treatment planning. A high affinity human ACE2 specific peptide, DX600, was selected and modified as a PET imaging agent for translational evaluation. Radiometal‐chelator conjugation did not affect binding nanomolar potency or specificity of the ligand, which was rapidly internalized. Preclinical investigation of ^68^Ga/^64^Cu‐HZ20 demonstrated ideal pharmacokinetic properties in models of human ACE2 expression, with rapid blood clearance, low background organ uptake and predominant renal clearance. High contrast of ACE2 pseudotumors was achieved within 2 h and within genetically engineered models.

After safety and dosimetric evaluation, ^68^Ga‐HZ20 was applied for a first‐in‐human translational study with 20 healthy volunteers of different ages and both sexes. To fully characterize this molecular imaging tool, dynamic scans were performed for each volunteer. The results showed highly consistent pharmacokinetics among the 20 volunteers, with high ACE2 expressing organs (such as kidney, gallbladder, testis for male) reflected in both high SUV_max_ and signal retention within the 3 h tested. The plateau of signal in expressing tissues and clearance from the blood pool afford convenient 90 min post‐administration static images useful to profile ACE2 expression.

Imaging results were mostly consistent with the HPA database (http://www.proteinatlas.org) and previous immunohistochemical profiling of ACE2, including microvilli of the intestinal tract and renal proximal tubules, gallbladder epithelium, testicular sertoli cells, and leydig cells (Table 1).^[^ ^16^, ^34^, ^50^ ^]^ Intriguingly, we report a differential finding between these imaging results and histological HPA data in the ACE2 expression in the female breast. We observed that the ACE2 expression level in breast is age dependent, with medium for young group and low for old group (*p* = 0.0002, Figure 6), while HPA reported no ACE2 expression.

PET imaging of ACE2 non‐invasively can provide real time and global receptor distribution quantitatively using the SUV_max_ value of the uptake of ^68^Ga‐HZ20 correlating directly with ACE2 expression level, which offers novel information relevant for infection by SARS‐CoV‐2, and pathology of COVID‐19. Recent reports have indicated that partial loss of the sense of smell or even total anosmia is early symptoms of SARS‐CoV‐2 infection.^[^ ^47^, ^48^ ^]^ It was suggested that the virus may exploit goblet and ciliated cells in the nasal epithelia as entry portals, a plausible primary infection site in many patients.^[^ ^51^ ^]^ Along the respiratory tract, the nasal mucosa showed higher ^68^Ga‐HZ20 uptake than oropharynx, and the lung showed the lowest uptake. It should be noted that the lower density of the lung tissue likely biases measured uptake values to underestimate tracer concentration. This observations are consistent with a recent study using high‐sensitivity RNA in situ mapping that has shown ACE2 expression is highest in the nose with decreasing expression throughout the lower respiratory tract, paralleled by a striking gradient of SARS‐CoV‐2 infection in proximal (high) versus distal (low) pulmonary epithelial cultures.^[^ ^52^ ^]^ This gradient could be clearly visualized in the same volunteer from the PET image.

Surprisingly, ^68^Ga‐HZ20 uptake in the lung and heart was very low. Data from the Human Protein Atlas and others has shown ACE2 receptor expression variability in patients bronchial and lung tissues with indications that underlying conditions inducing inflammation^[^ ^63^ ^]^ may have a role in expression levels.^[^ ^53^ ^]^ An interesting finding from these comparisons across patients in the imaged cohort is that there are negligible differences between young and geriatric males or females. These data potentially underline the equivalent risk of infection across all adults, and that further evaluation can be accomplished with the tracer noninvasively.

Studies have found that 70% of patients infected with SARS‐CoV accompanied by diarrhea. A recent case report described that ACE2 is highly expressed in stratified epithelial cells and absorptive intestinal cells in the upper esophagus, ileum, and colon.^[^ ^54^ ^]^ These findings may support the possibility of fecal‐oral transmission route, and also consistent with the uptake of ^68^Ga‐HZ20 in the oropharynx, intestine, and rectum observed in our imaging results (Table 1).

Several of our results highlight expression profiles in reproductive organs. Recent studies have reported testicular damage caused by SARS‐Cov‐2 infection,^[^ ^46^, ^55^ ^]^ and investigation by PET on reproductive function of recovered male patients, especially youth^[^ ^35^ ^]^ is warranted. Besides ACE2 expression observed in the breast of young female, ^68^Ga‐HZ20 uptake was observed in ovaries (without a difference between young and old women). We also found high ^68^Ga‐HZ20 uptake in corpus luteum in two young women, but not in immature follicles. ACE2 expression in antral follicles, mature luteal cells, the theca and stromal cell layer in ewes has been previously described,^[^ ^56^ ^]^ but to our knowledge this is the first observation of ACE2 in human corpus luteum with the direct comparison around surrounding organs. The ACE2 specific inhibition of the cyclic DX600 peptide has been established, however uptake at these unexpected sites may suggest non‐specific interactions which can be definitively determined with future biopsy confirmation.

Acute kidney injury has also been commonly found in patients infected with SARS‐CoV‐2,^[^ ^57^ ^]^ with likely long term impacts on overall health. In addition to the host's immune response, these acute sequelae of fighting the infection may also come from the direct attack of the virus on the target cells expressing ACE2.^[^ ^58^ ^]^ RNA‐Seq studies have shown that ACE2 is abundantly expressed in various renal proximal tubule cell subtypes; consistent with our observations. Limited by qualified resources for carrying out studies in patients during the SARS‐CoV‐2 infection, we were only able to image one recovered volunteer in which we report dramatic changes in observed ACE2 expression (Figure 3D and Figure S9, Supporting Information). We stress that conclusions cannot be drawn across healthy volunteers and the recovered patient. However, these data strongly motivate further investigations in a larger recovered patient pool.

In summary, we have developed a non‐invasive imaging method using ^68^Ga/^64^Cu‐HZ20 PET to interrogate the global expression profile of ACE2 in human, the key receptor for SARS‐CoVs to infect human cells. To our knowledge, this is the first study to quantitatively profile ACE2 expression by PET imaging, and this approach demonstrates the capacity to measure organ express profiles at baseline and following COVID‐19. The results from 20 healthy volunteers were consistent with pathological reports of receptor distribution, while preliminary imaging data from a SARS‐CoV‐2 infection recovered man may suggest ACE2 level changes. These preliminary results require further investigation in a wider patient population. With the capacity of quantitatively detecting stable and transient ACE2 expression non‐invasively, this quantitative imaging method may have utility to evaluate differences across patients for SARS‐CoV‐2 infectivity; COVID‐19 symptom severity and duration; and for evaluation of physiological effects from other emerging and novel coronaviruses.

## 4 Experimental Methods

### Ethical Statement

Human study was approved by the Ethics Committee of Peking University Cancer Hospital (2020KT62) and registered in Chinese Clinical Trial Registry (ChiCTR2000033675, Date of registration: June 8, 2020) and ClinicalTrials.gov (NCT04422457, Date of registration: June 9, 2020). All clinical study was conducted following the latest guidelines of the Declaration of Helsinki. All animal studies were performed according to a protocol approved by the Peking University Cancer Hospital Animal Care and Use Committee and the Department of Comparative Medicine at the Washington University in St. Louis School of Medicine (Protocol Number 20 190 006 and 20–0183).

### General Procedures

All solvents and chemicals purchased from commercial sources were of analytical grade or better and were used without further purification. DX600, NODAGA‐DX600, and DOTA‐DX600 were custom synthesized by ChinaPeptides Co., Ltd (Shanghai, China) or CSBio (San Diego, California). Sep‐Pak Accell Plus QMA and Sep‐Pak C18‐Light cartridges were purchased from Waters (Ireland). Acrodisc 25 mm syringe filter (0.22 µm) was purchased from Pall Corporation (USA). The product was analyzed by radio‐ high performance liquid chromatography (HPLC) (1200, Agilent, USA) equipped with *γ* detector (Flow‐count, Bioscan, Washington. D.C., USA), using a C18 column (Eclipse Plus C18, 4.5 × 250 mm, 5µm, Agilent, USA). The product purity was also determined using Radio‐TLC (AR 2000, Bioscan, USA) after radiolabeling. The PET/CT imaging studies of small animals were performed on the Mira PET/CT of PINGSENG Healthcare Inc. (Shanghai, China), or microPET R4 rodent scanner (Siemens) and analyzed by ASIProVM. The Clinical PET/CT scans were obtained on a Biograph mCT Flow 64 scanner (Siemens, Erlangen, Germany) with unenhanced low‐dose CT. ^68^Ga‐HZ20 PET/MRI was performed on a hybrid 3.0T PET/MR scanner (uPMR790, UIH, Shanghai, China) in female volunteers.

### Radiolabeling and Quality Control of Radiopeptides

*^68^Ga‐HZ20*: 195 µL 1.0 m NaOAc solution containing 40 µg (1.17 × 10^−5^ mmol) DOTA‐DX600 was added into 3.0 mL of ^68^GaCl_3_ freshly eluted from ^68^Ge–^68^Ga generator (Isotope Technologies Garching, Germany) by 0.05 m of hydrochloric acid, with the radioactivity ranging from 370 to 1110 MBq. The final pH of the reaction was controlled as 4.2 and the mixture was heated at 95 °C for 15 min. After the reaction completed which was monitored by radio‐TLC, the mixture was loaded onto an activated Sep‐pak C18 cartridge. The cartridge was first washed with 5 mL of water to remove the free ^68^Ga, and then eluted with pure ethanol to obtain the product of ^68^Ga‐HZ20 as a 0.5 mL ethanol solution. The ethanol solution of the product was diluted with 10 mL 0.9% saline and filtered through a 0.22 µm filter (Merk, Darmstadt, Germany) to make the radiopharmaceutical used in this study. It contains 37–370 MBq of ^68^Ga‐HZ20 in ethanol‐water (ethanol < 5%).

The solution was analyzed by radio‐HPLC to assess the radiochemical purity. The HPLC was eluted with water‐CH_3_CN system (Phase A: 0.1% TFA H_2_O; Phase B: 0.1% TFA CH_3_CN) using gradient elution (0–5 min 20% B; 5–10 min 20%–80% B; 10–12 min 80% B; 12–15 min 80%–20% B) at a flow rate of 1.0 mL min^−1^. The radiolabeling yield was 59.9 ± 3.9% (non‐decay corrected, *n* = 10) and the radiochemical purity was over 95%. In vitro stability study of ^68^Ga‐HZ20 in phosphate buffer saline was performed by adding 50 µL of ^68^Ga‐HZ20 to 450 µL of phosphate buffer saline and incubating at 37 °C. At 1, 2, and 4 h time points, 10 µL aliquot was analyzed by radio‐HPLC to assess the radiochemical purity.

*^64/nat^Cu‐HZ20*: ^64^Cu‐labeled NODAGA‐DX600 was prepared by dissolving 20–40 µg of peptide in 10–20 µL of trace‐free water and 50 µL of 0.25 m ammonium‐acetate buffer (pH 6.0), and adding 37–74 MBq ^64^CuCl_2_ solution (1–2 µL) followed by a 5 min incubation at 95 °C. After incubation, the final product was analyzed with analytical HPLC (column: Kromasil 100‐5‐C18, 4.6 × 150 mm; flow rate: 1.0 mL min^−1^; mobile phase: 0.1% TFA in water and CH_3_CN; gradient: 0–10 min, 10%–50% CH3CN; 11–14 min, 95% CH_3_CN, 15 min, 10% CH_3_CN), and reformulated with PBS/BSA (1.0% bovine serum albumin) solution for further experiments. If needed, purification with HLB cartridge (Phenomenex; Torrance, CA). For binding studies, one equivalent of ^nat^Cu(SO_4_)_2_ was further added into reaction mixture, and the final solution was incubated for another 5 min to generate structurally identical ^64/Nat^Cu‐labeled HZ20 for saturation binding studies.

### Surface Plasmon Resonance Assay

The binding affinity between DX600 (or DOTA‐DX600) and ACE2 was determined by SPR assay with Nicoya Open SPR system (Nicoya Lifesciences Inc., Ontario, Canada), in comparison to DX600. Briefly, recombinant human ACE2 protein (50 µg mL^−1^; BP003061) was immobilized on the surface of the nanogold sensor chip, after the chip was activated by 1‐ethyl‐3‐(3‐dimethylaminopropyl)carbodiimide/*N*‐hydroxysuccinimide. Various dilutions of DX600 or DOTA‐DX600 were added at a flow rate of 20 µL min^−1^ for 7 min and the SPR signal were collected. *K*_D_, ka and kd were calculated using Trace Drawer Evaluation version 2.0 (Trace Software International, Saint‐Romain, France).

### Saturation Affinity of ^64/Nat^Cu‐HZ20 Binding to hACE2

Saturation affinity studies were performed in HEK293‐hACE2 cells and HEK293‐WT using various concentrations of ^64/Nat^Cu‐HZ20. Triplicate samples containing 0.28 × 10^6^ cells and 0.01–2000 nmol of ^64/Nat^Cu‐HZ20 in 0.25 mL cell culture medium were incubated at 37 °C for 1 h and 4 °C for 24 h, respectively. The cells were collected with glass microfiber filters, washed with 4 × 2 mL of ice‐cold TBS (pH 7.4), and radio‐assayed with a gamma counter. ^64/Nat^Cu‐HZ20 uptake (molecules per cell) in the HEK293‐hACE2 cells was plotted versus ^64/Nat^Cu‐HZ20 concentration, and Kd values were estimated using a least‐squares fitting routine (GraphPad Prism 8, San Diego, CA).

### In Vitro Uptake of ^64^Cu‐HZ20

hACE2‐positive cells (HEK293‐hACE2) and HEK293‐WT cells were cultured in DMEM HG cell medium and pre‐seeded in 6‐well plate the day before cell uptake experiments. Cells (0.1 × 10^6^ in a total volume of 0.95 mL cell medium) in triplicate were mixed with ≈26 kBq of ^64^Cu‐HZ20 in 0.05 mL PBS buffer (final concentration: 2.5 nm), and the cell mixtures were incubated at 37 °C for 1 h. Nonspecific uptake was determined by co‐incubating with 10 µg of DX600. After incubation, the unbound radioactivity was collected and then the cells were washed twice with cold PBS. Glycine buffer (pH 2.8) was used to incubate the cells for 5 min over ice bath two times to remove the non‐internalized radiotracer. After incubation, the cells were destroyed with 1 m NaOH and the residual was collected for measuring the internalized radiotracer. For kinetic uptake studies, the samples were incubated at 37 °C for 15, 30, 60, and 120 min and the cells were measured with a gamma‐counter.

### PET Imaging in Animals

*^68^Ga‐HZ20*: Four‐week‐old female athymic nude mice were purchased from SLAC Laboratory Animal Co. Ltd., China. 5 × 10^6^ of HepG2 cells were inoculated into the right front shoulder region of the mice to build the xenograft tumor model with ACE2 expression. When the tumor volumes were estimated to be 450–800 mm^3^, the mice were used for the studies. Under isoflurane anesthesia, mice bearing HepG2 tumors (*n* = 4 per group) were injected intravenously with 100 µL of 3.7 MBq ^68^Ga‐HZ20 for small animal PET. Scans were performed at 60, 120, and 180 min after administration. Unlabeled DX600 (50 mg kg^−1^ body weight) and 3.7 MBq ^68^Ga‐labeled tracers were co‐injected into mice bearing HepG2 tumors (*n* = 4 per group) for blocking study as a control group. Subsequently, animals were scanned at 60, 120, and 180 min post‐injection. Regions of interest were defined over tumor, heart, liver, lung, kidney, and muscle and uptake computed relative to the injected activity.

Female immunocompromised R2G2 mice from Jackson Laboratories (Bar Harbor, Maine) were used for subcutaneous inoculation of dual pseudotumors of hACE2 and control (non‐expressing) HEK293T cells. The cells (3 × 10^6^) suspended in a 1:1 mixture of PBS and Matrigel were implanted on the shoulder area. Tumors were monitored and imaging commenced after reaching >200mm^3^.

Dual xenografts‐bearing mice or transgenic mice were injected with about 3.0 MBq (0.3 nmole) of ^64^Cu‐HZ20 via tail vein injection. At 1, 4, and 20 h post‐injection, PET imaging was performed for 15 or 30 min on microPET R4 rodent scanner (Siemens) with the tumors centered in the field of view, and the animal under 2% isoflurane anesthesia. PET images were reconstructed by an iterative 3D maximum a priori algorithm. The calibration factor of the PET scanner was measured with a mouse‐sized phantom composed of a cylinder uniformly filled with an aqueous solution of ^18^F with a known activity concentration. ROI analysis of the acquired images was performed using ASIProVM (Siemens) and the observed percent injected activity of tissue (%IA mL^−1^) was measured.

Following PET imaging, tumors were excised, embedded in optimal‐cutting‐temperature mounting medium (OCT, Sakura Finetek), and frozen on dry ice prior to cutting a series of 10 µm frozen sections. Digital autoradiography was performed on a phosphor imaging plate at −20 °C. Phosphor imaging plates were read at a pixel resolution of 40 µm with a Cyclone system (Perkin Elmer). Following autoradiography imaging, the same section was stained with H&E and whole‐mount bright field images acquired in a similar manner.

The dual xenografts‐bearing animals were randomly assigned into three cohorts and administrated ≈3.0 MBq (0.3 nmole) of ^64^Cu‐HZ20. At 1, 4, and 20 h post‐injection, the animals were sacrificed for tissue dissection. The organs of interest were collected, rinsed, blotted, weighed, and counted with a *γ*‐counter (Perkin Elmer Wizard^2^). The total injected radioactivity per animal was determined from the measured radioactivity in an aliquot of the injectate. Data were expressed as percent of injected activity per gram of tissue (%IA g^−1^).

### Preclinical Immunohistochemistry

Harvested mouse tissues were formalin‐fixed, paraffin‐embedded, and sliced at 4 µm thickness. Normal human tissue blocks were acquired from the tissue bank at the Peking University Cancer Hospital Pathology Department Central Laboratory. Sections were incubated with 3% H_2_O_2_ at RT for 10 min. Antigen was retrieved from the tissue in citric acid buffer (0.01 m, pH 6) with microwave heating for 2.5 min, followed by a 5 min cooling step. For normal human tissues, microwave heating was extended to several rounds of 3 min exposure. Tissues were blocked with goat antiserum for 30 min, stained with rabbit anti‐ACE2 antibody (1:100 ab108252, Abcam) at 4 °C overnight. The washed sections were then stained with goat anti‐rabbit secondary (PV‐6001, Beijing Zhongshan Jinqiao Biologicals) for 30 min at RT. Subsequently, the sections were developed with 3, 3′ diaminobenzidine tetrahydrochloride (DAB), dehydrated in an alcohol gradient, and sealed in neutral mounting media, and scanned (Aperio Versa 200, Leica).

### Clinical ^68^Ga‐HZ20 Release

68Ga‐HZ20 was prepared as above under GLP environment dispensing hot cell (NMC Ga‐68, Tema Sinergie, S.p.A, Italy). The radiotracer met or exceeded release quality control and criteria (Table S1, Supporting Information). Patients received tracer at a mean administered activity of 2.48 MBq kg^−1^ (±0.42 MBq kg^−1^).

### Imaging Study Subjects Enrollment

The informed consent was obtained from the volunteers for publication. The inclusion criteria for healthy subjects included: 1) older than 18 years, 2) the ability to provide informed written consent, 3) a medical history without any significant comorbidities, including physical examination, electrocardiogram, hematology, and biochemistry. The exclusion criteria included: 1) liver and renal function dysfunction, 2) pregnancy or current lactation. Following the criteria, four groups of subjects (five male with age below 50, four male with age above 50, five female with age below 50 and six female with age above 50) were enrolled in this study, for a total of 20 subjects. The age ranged from 32 to 72 with a mean of 51.1 ± 15.1 and the demographic data of all the volunteers were shown in Table S2, Supporting Information.

### PET/CT Examination Procedures

No specific preparation was required for the subjects on the day of ^68^Ga‐HZ20 PET scanning. A low‐dose CT scan (120 kV, 35 mA, slice 0.6 mm, matrix 512 × 512) was per‐formed before the ^68^Ga‐HZ20 injection. Then, a whole‐body dynamic PET scan was performed immediately after the intra‐venous injection of ^68^Ga‐HZ20 in all subjects and continued for ≈40 min (five passes, round 10 min for each pass). All the subjects also underwent static whole‐body PET/CT scan at 90 and 180 min post‐injection. Dynamic whole‐body PET/CT scans were performed on a Biograph mCT Flow 64 scanner (Siemens, Erlangen, Germany) with the setting of 120 kV, 146 mAs, slice 3 mm, matrix 200 × 200, full width at half maximum (FWHM) 5 mm, filter: Gaussian, field of view (FOV) 256 (head), 576 (body). The patient bed was set to continuously move at a speed of 2 mm s^−1^ to cover the entire body of each subject (from the top of the skull to the middle of the femur). Static whole‐body PET/CT scan used a speed of 1 mm s^−1^ at 90 min and 0.8 mm s^−1^ at 180 min. 3D iterative reconstruction was applied for image reconstruction, with CT‐based attenuation and scatter correction through standard vendor‐based reconstruction. The ^68^Ga activities were decay corrected to the time of injection and normalized to the total activity administered.

### PET/CT Image Analysis

MultiModality Workplace (Siemens, Erlangen, Germany) was used for data processing. To analyze the biodistribution of ^68^Ga‐HZ20, regions of interest (ROIs) were manually drawn on the largest transverse section of major organs/tissues, while avoiding major blood vessels. The normal organs/tissues selected for VOI analysis included: the brain, parotid glands, nasal mucosa, oropharynx, nasopharynx wall, thyroid, cardiac muscle, left heart ventricle, lungs, liver, gallbladder, pancreas, spleen, kidneys, red marrow, bone, stomach, small intestine, upper and lower colon, rectus, breast, uterus, ovary, prostate, testis, and muscle (quadriceps femoris). The maximum single‐voxel standardized uptake value (SUV_max_) were generated automatically from ROIs in MultiModality workplace for analysis and comparison. SUV_max_ is defined as:

(1) $$\mathrm{SU}V_{\max}=r/\left(\frac{a^{\prime }}{w}\right)$$

where *r* is the maximum radioactivity activity concentration (kBq mL^−1^) measured by the PET scanner within the defined ROI, *a*′ is the decay‐corrected amount of injected radiolabeled ^68^Ga‐HZ20 (kBq), and *w* is the weight of the patient (g).

### Statistics

Statistical analyses were performed with SAS software (V.9.4, SAS) and Prism (V8.0, GraphPad Software). The organ uptake data in the form of SUV_max_ were grouped by gender and by age. To compare distributions among samples, the parametric continuous variables were expressed as mean ± SD. A mixed model was applied to compare the different groups for different time points, with study subjects as random effects. Independent sample *t*‐tests were used to compare SUV_max_ values between different groups. A *p*‐value less than 0.05 was considered to be significant.

## Conflict of Interest

The authors declare no conflict of interest. Intellectual property protection has been filed by Washington University in St. Louis School of Medicine and Ha.Z and D.T.

## Author Contributions

Hu.Z., Ha.Z., and N.Z contributed equally to this work. Hu.Z., Ha.Z., D.T., X.Y., and Z.Y. conceived and designed the experiments. J.D., Ha.Z., and Z.L. radio‐synthesized and characterized the agent. J.D., N.B., and Z.L. conducted preclinical research and quality control of radio‐tracer. N.Z., Ha.Z., N.B., J.J., and D.T. performed the PET, PET/CT, and PET/MRI studies. N.B., and Q.Z. performed the immunohistochemistry and dose calculations. N.Z., Hu.Z., Ha.Z., N.B., T.L., H.J., and L. L. analyzed the data. X.Y., Hu.Z., D.T., and Ha.Z. co‐wrote the paper. Z.L. and Z.Y. provided constructive discussion. All authors discussed the results and analysis and commented on manuscript.

## Supporting information

Supporting Information

Supplemental Video 1

Supplemental Video 2

Supplemental Video 3

## Data Availability

Research data are not shared.
