# Supplementary material for: Molecular PET/CT Profiling of ACE2 Expression In Vivo: Implications for Infection and Outcome from SARS‐CoV‐2
Source: Adv Sci (Weinh). 2021 Jun 26;8(16):2100965. doi: 10.1002/advs.202100965 (PMC8373167; doi:10.1002/advs.202100965)
Supplement: Supplementary file 1 — Supporting Information [file ADVS-8-2100965-s004.pdf]

© 2021 Wiley-VCH GmbH

ADVANCED SCIENCE
Open Access

Supporting Information

for *Adv. Sci.,* DOI: 10.1002/advs.202100965

Molecular PET/CT profiling of ACE2 expression in vivo: implications for infection and outcome from SARS-CoV-2

Hua Zhu, Hanwen Zhang, Nina Zhou, Jin Ding, Jinquan Jiang, Teli Liu, Ziyu Liu, Feng Wang, Qian Zhang, Zhuochen Zhang, Shi Yan, Lei Li, Nadia Benabdallah, Hongjun Jin, Zhaofei Liu, Lisheng Cai, Daniel L.J. Thorek*, Xing Yang*, and Zhi Yang**

Supplementary Materials:

List of Supplementary Materials:

Supplemental Figure 1 – Mass spectra

Supplemental Figure 2 – In vitro binding results

Supplemental Figure 3 – Preclinical uptake and imaging

Supplemental Figure 4 – HPLC and radiochromatogram data

Supplemental Figure 5 – Body weight change with HZ20 dosing

Supplemental Figure 6 – Biodistribution of ^{68}Ga-HZ20

Supplemental Figure 7 – HepG2-hACE2 targeting data

Supplemental Figure 8 - Summary of clinical imaging by SUVmax

Supplemental Figure 9 – Comparison of SUVmax in healthy volunteers and recovered patient

Supplemental Figure 10 – Dynamic imaging series for #002

Supplemental Figure 11 – Conjuctiva uptake by PET/CT

Supplemental Figure 12 – Breast SUVmax by age

Supplemental Figure 13 – Bilateral ^{68}Ga-HZ20 uptake comparison

Table S1 - Quality control of ^{68}Ga-HZ20 used in this study

Table S2 - General information of volunteers enrolled in this study.

Movie S1

Movie S2

Movie S3

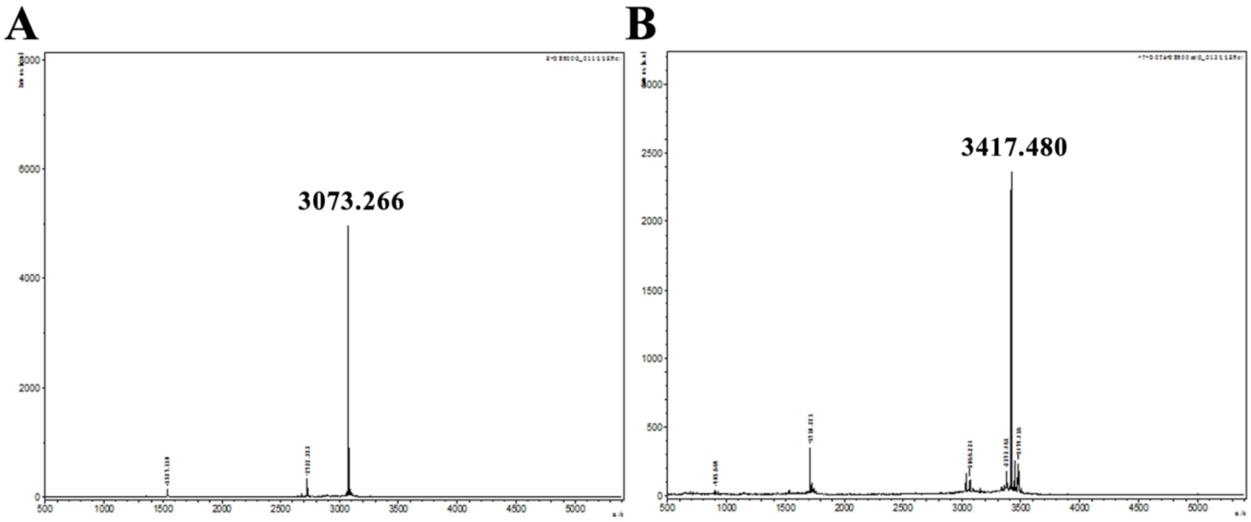

Supplemental Figure 1. The mass spectrum of DX600 and DOTA-DX600. (**A**) MALDI-TOF mass spectrum of DX600, Cal. M-H- [C141H186N35O40S2-] = 3073.31, m/z = 3073.26. (**B**) MALDI-TOF mass spectrum of DOTA-DX600, Cal. M-H- [C155H210N39O46S2-] = 3417.47, m/z = 3417.48.

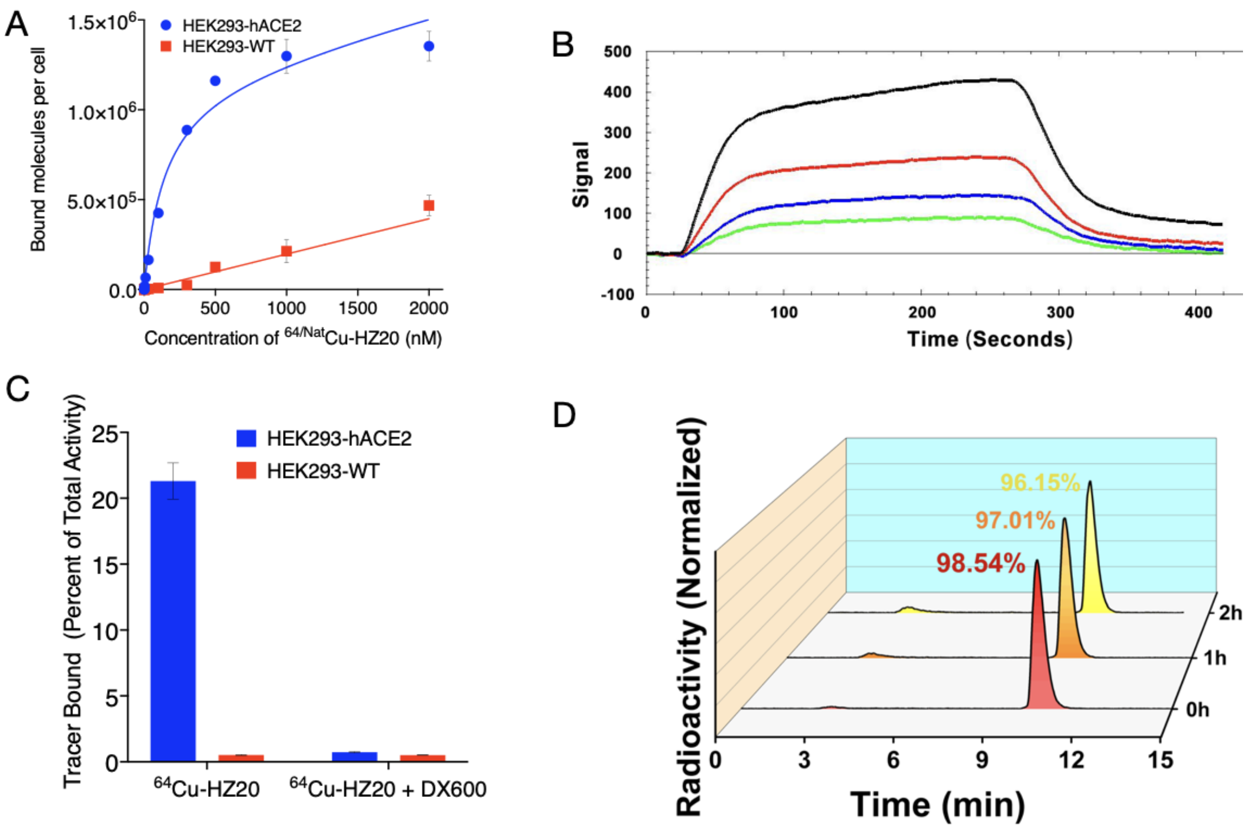

Supplemental Figure 2. In vitro evaluation of ^{64}Cu-HZ20 and ^{68}Ga-HZ20. (**A**) Saturation binding assay of $^{64/Nat}$Cu-HZ20 over HEK293-hACE2 and HEK293-WT(non-transduced) at 37 °C. (**B**) binding assay of DX600. (**C**) hACE2 expression specifically determine the accumulation of ^{64}Cu-HZ20 over HEK293-hACE2 and HEK293-WT cells for 2 h incubation at 37 °C. (**D**) Stability analysis of ^{68}Ga-HZ20 over time in 0.01 M PBS solution at 37°C.

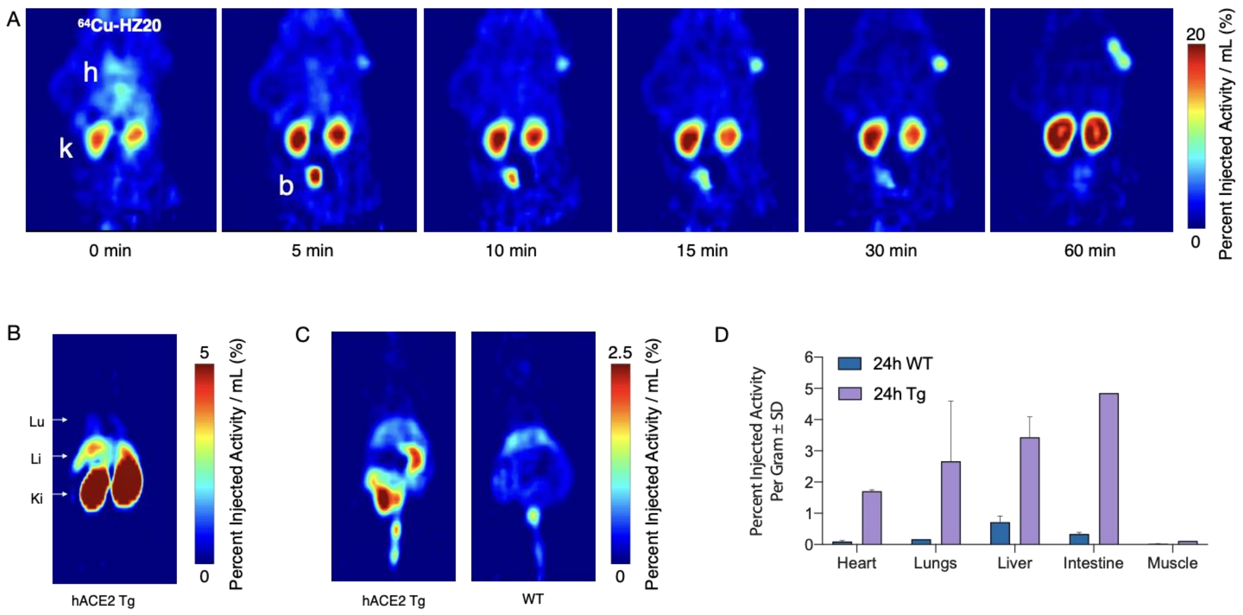

Supplemental Figure 3. Dynamic PET imaging of the dual xenografts and hACE2 transgenic mice with ^{64}Cu-HZ20. (**A**) Dynamic imaging of HEK293-hACE2 (right) and HEK293-WT(left) for 60 min after dose administration. (**B**) Static imaging of hACE2 transgenic(Tg) mice at 2 h post injection. (**C**) Comparable PET imaging of hACE2 Tg and wild type animal at 24 post injection. (**D**) Quantitative analysis of static ^{64}Cu-HZ20 imaging of hACE2 Tg and wild type mice, 24 h post injection.

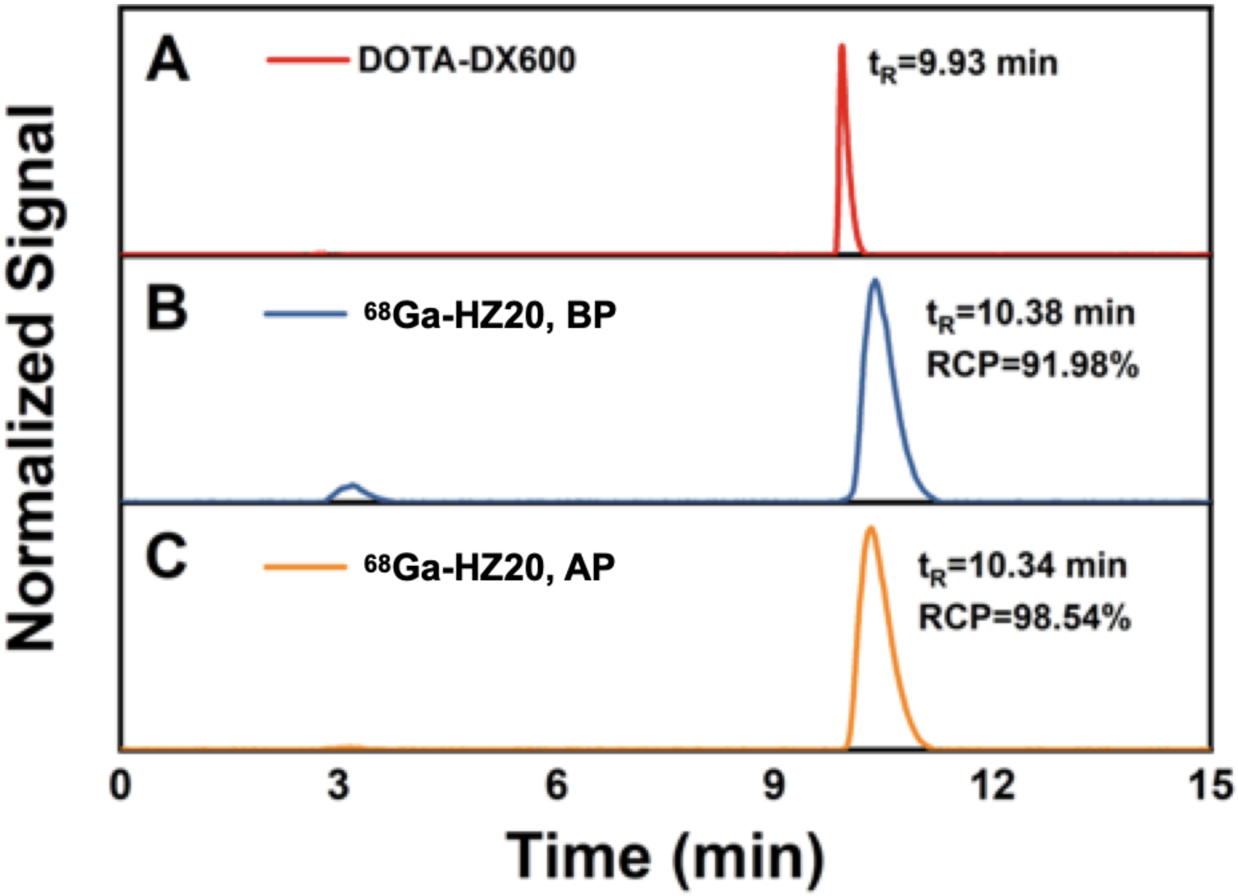

Supplemental Figure 4. The HPLC results of HZ20 radio-labeling. (**A**) The HPLC signal of HZ20 monitored by UV channel, t_R = 9.93 min. (**B**) The HPLC signal of ^{68}Ga-HZ20 before purification monitored by radioactivity channel, t_R = 10.38 min. The calculated radio-chemical purity was 91.98%. (**C**) The HPLC signal of ^{68}Ga-HZ20 after purification monitored by radioactivity channel, t_R = 10.34. The calculated radio-chemical purity was 98.54%. The HPLC was eluted with water-CH_3CN system (Phase A: 0.1% TFA H_2O; Phase B: 0.1% TFA CH_3CN) using gradient elution (0-5 min: 20% B; 5-10 min: 20%-80% B; 10-12 min: 80% B; 12-15 min: 80%-20% B) at a flow rate of 1.0 mL/min.

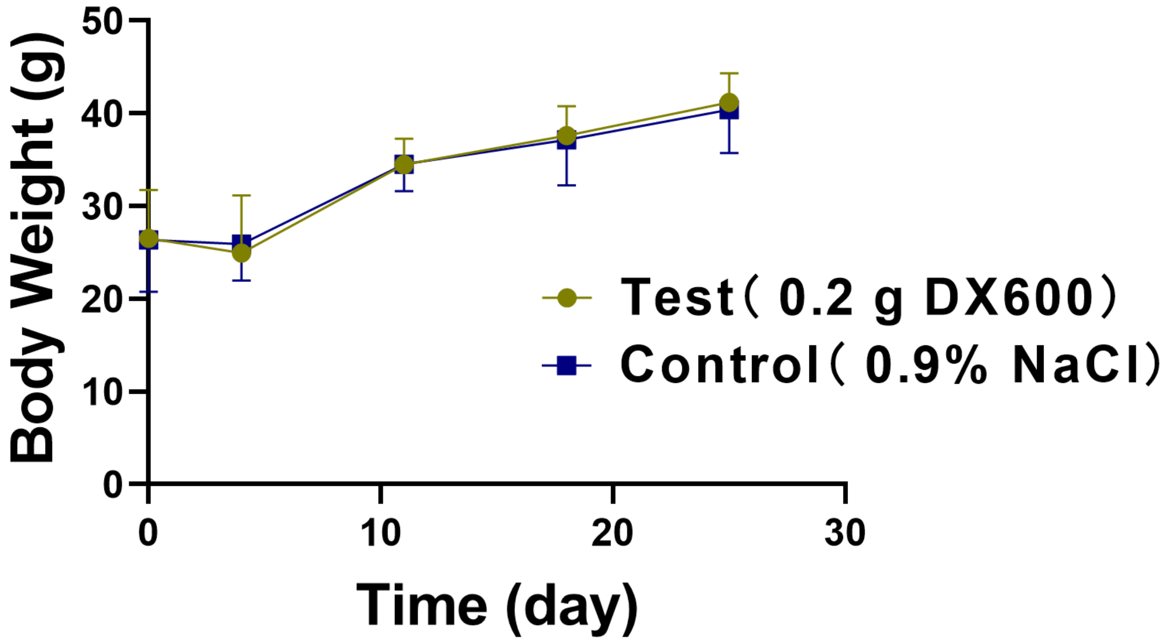

Supplemental Figure 5. Body weight monitoring following DX600 exposure was conducted in mice for 25 days. The experiment was carried out in 10 mice at 4-weeks of age. The mice were either injected with DX600 of 200 μg/200 μL or 0.9% normal saline of the same volume. The injection was repeated on day 0, day 4, day 11, day 18 and day 25, and the weight of the mice was recorded. The results showed that there was no significant difference between the experimental group (n = 5, weight from 26.5 ± 5.2 g to 41.2 ± 3.1 g) and the control group (n = 5, weight from 25.4 ± 5.6 g to 40.4 ± 4.7 g).

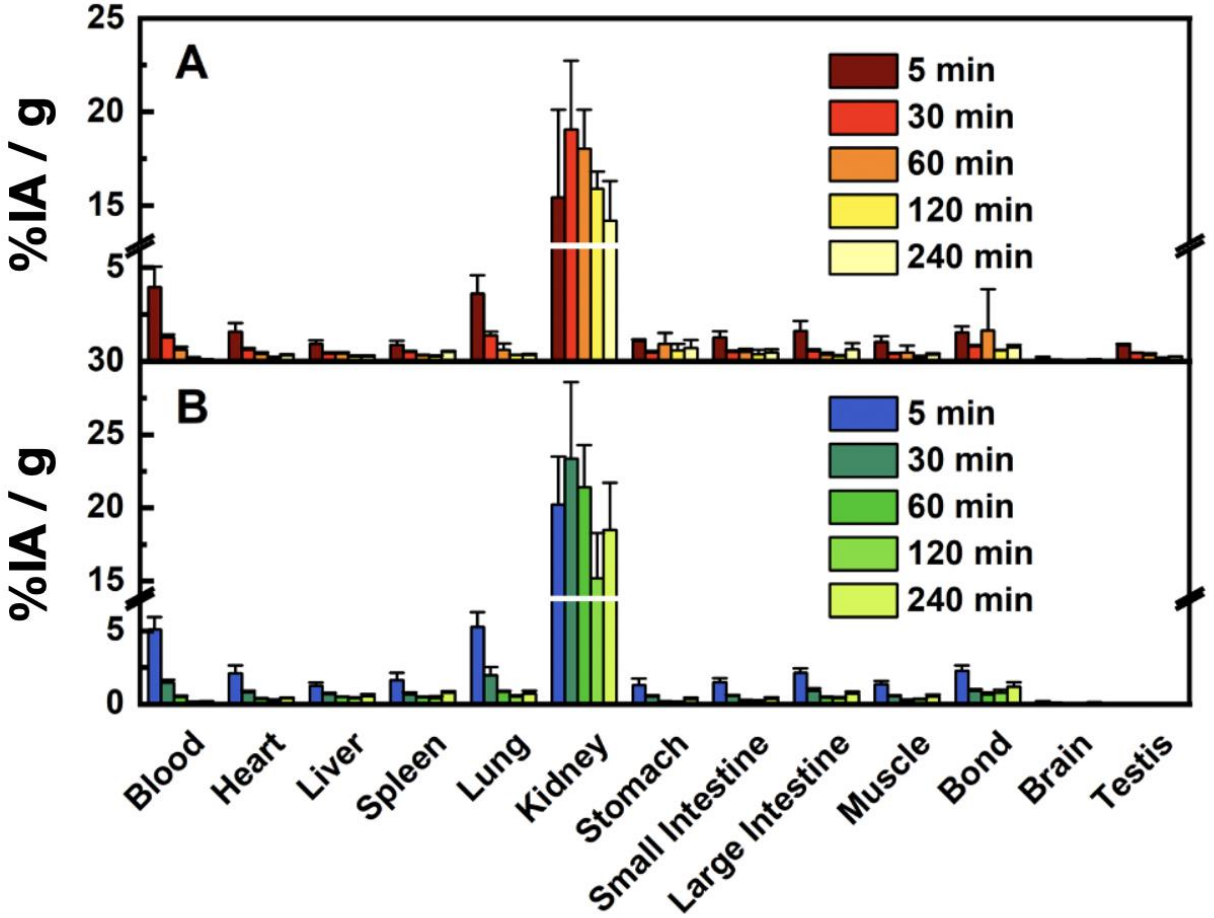

Supplemental Figure 6. Biodistribution of ^{68}Ga-HZ20 in normal male/female mice. (**A**) Bio-distribution results in male mice at 5, 30, 60, 120, 240 min post-injection (n = 4). (**B**) Bio-distribution results in female mice at 5, 30, 60, 120, 240 min post-injection (n = 4). The distribution results showed that kidneys received the highest radioactivity uptake and the radioactivity could also accumulate in the spleen, stomach, intestine and several other tissues. The normal animal body distribution was used for preliminary analysis and reference before translation research. Data shown as percent injected activity per gram of tissue (%IA/g).

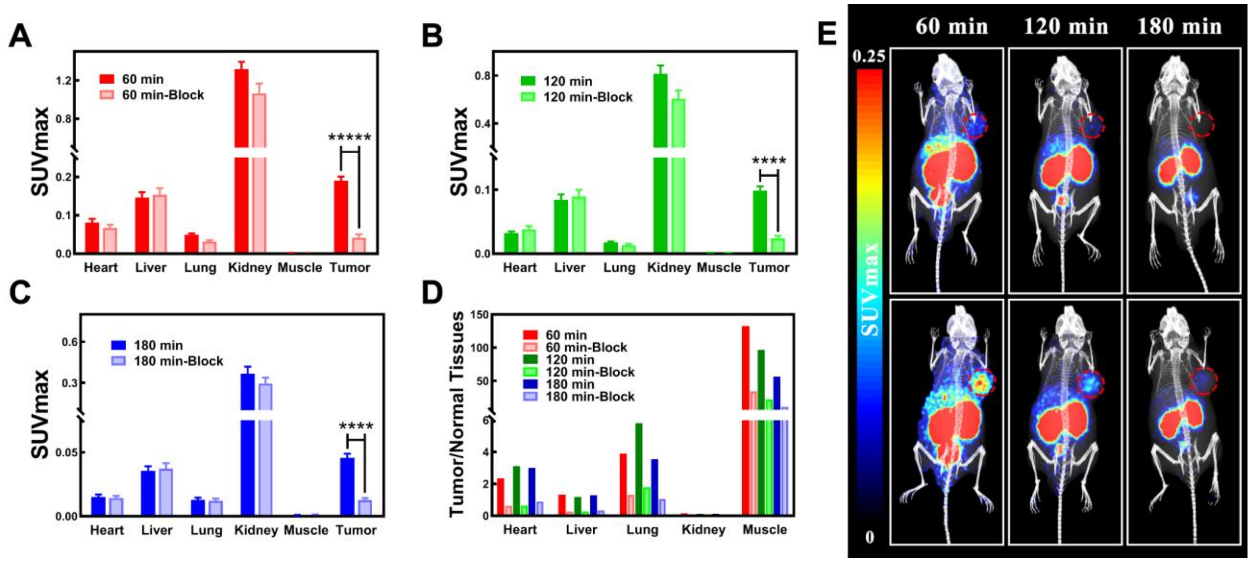

Supplemental Figure 7. Micro-PET imaging and quantitative analysis of ^{68}Ga-HZ20 in the HepG2 tumor bearing mice. Comparison of the maximum standardized uptake value (SUVmax) of each organ between the experimental group and the blocking control group at (**A**) 60 min, (**B**) 120 min and (**C**) 180 min. Tumor, heart, liver, lung, kidney and muscle were selected for analysis. (**D**) The ratio of the uptake of tumor-to-normal-organ between experimental group and blocking group at each time point. (**E**) Comparison of images between the experimental group and blocking control under the same scale, with the control showed on the top row. ^{68}Ga-HZ20 has a specific uptake in HepG2 tumors expressing human ACE2, which is significantly higher than that of the blocking group (****: $0.00001<P\leq0.0001$, *****: $P\leq0.00001$). DX600 was applied as blocking agent with a dose of 50mg/kg.

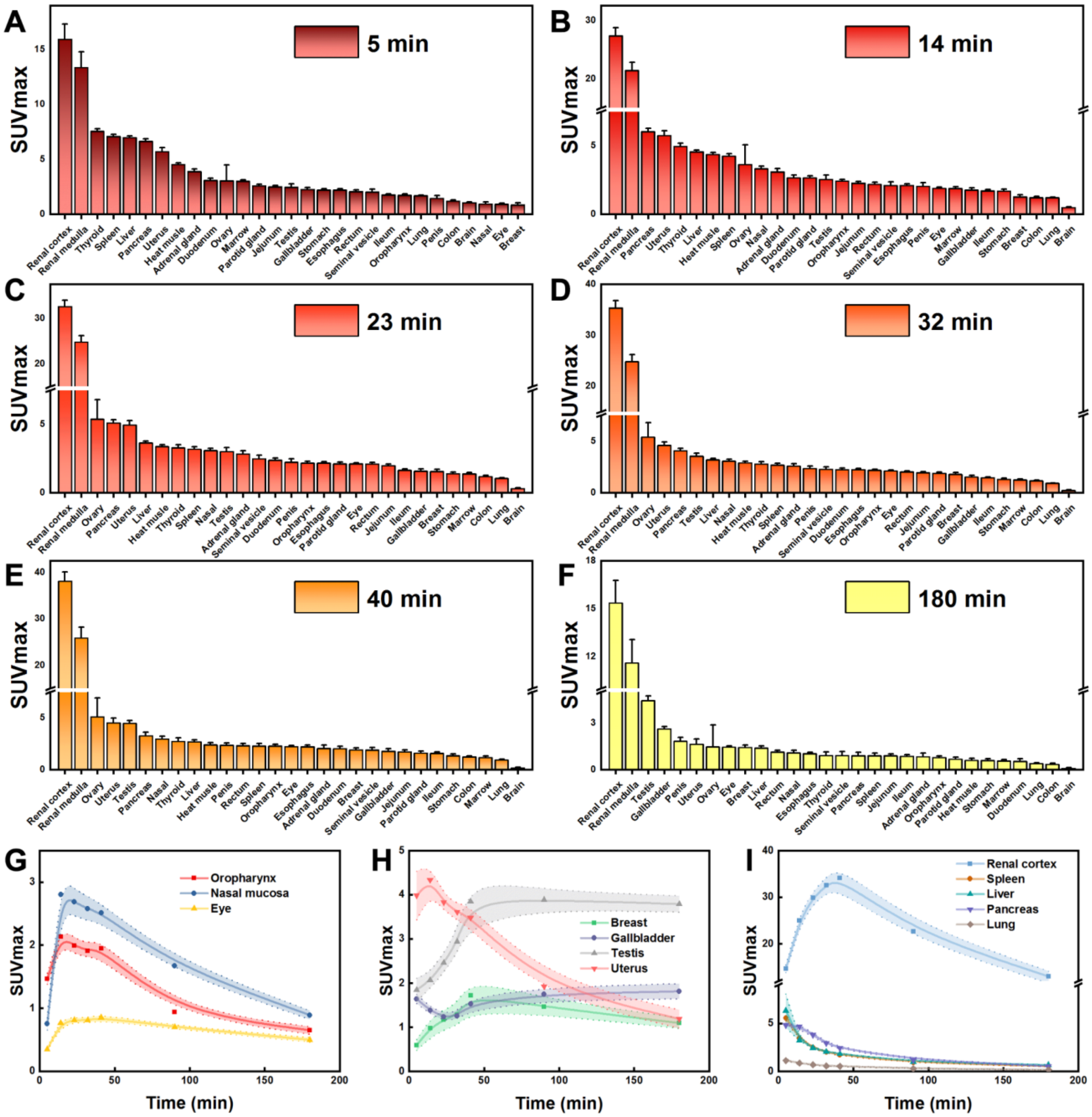

Supplemental Figure 8. The summary of SUVmax results in 20 volunteers at different time points. The SUVmax distribution at (**A**) 5 min, (**B**) 14 min, (**C**) 23 min, (**D**) 32 min, (**E**) 40 min, (**F**) 180 min. The dynamic changes of SUVmax in typical organs (**G**), (**H**) and (**I**).

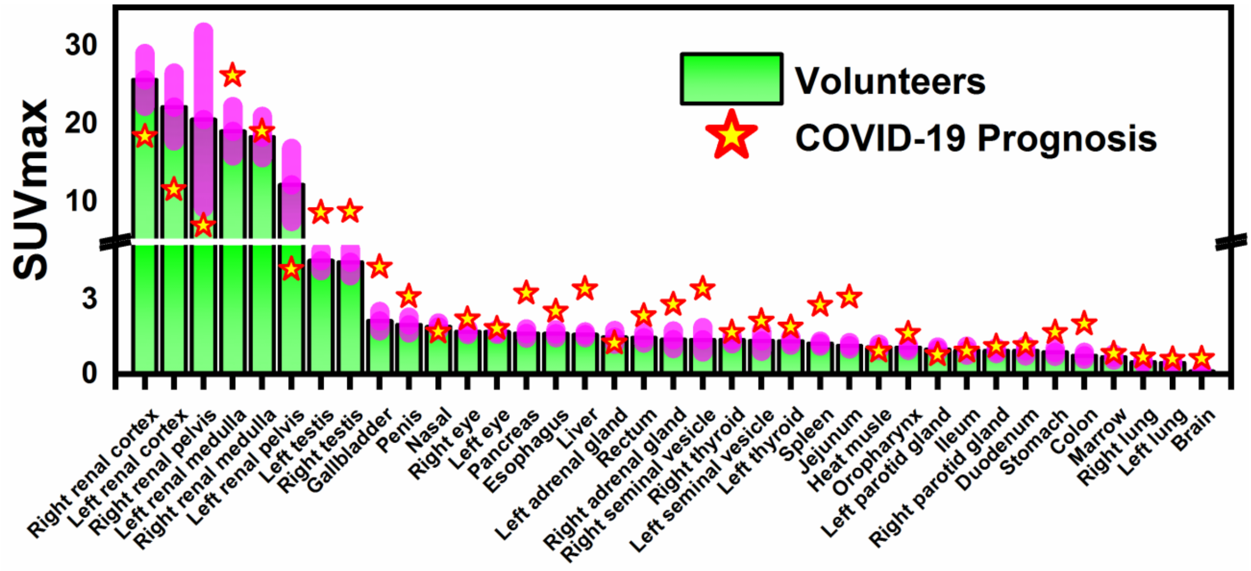

Supplemental Figure 9. The uptake in most organs of the recovered COVID-19 patient at 90 min were not within the confidence interval of healthy volunteers, except for the right renal medulla, left adrenal gland and ileum. There is a considerable difference between the recovered patient and healthy volunteers.

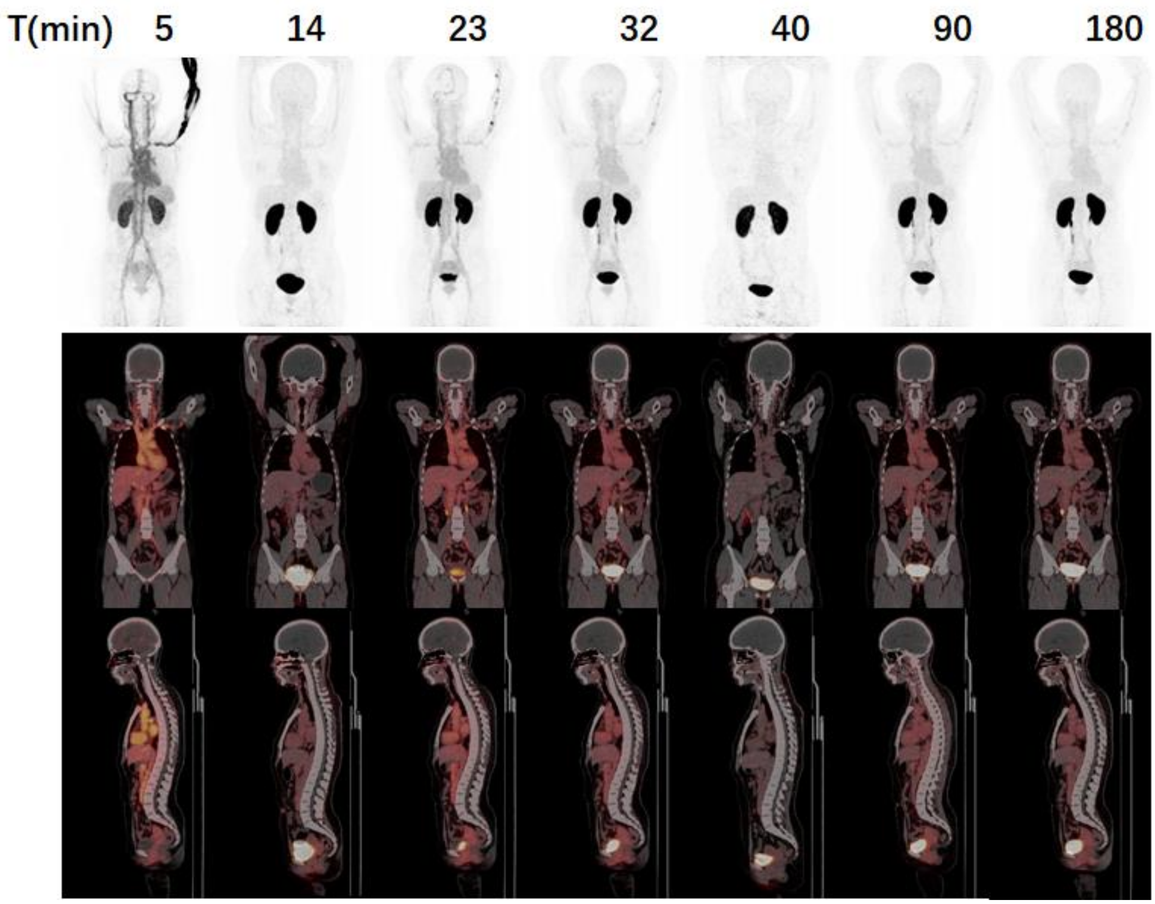

Supplemental Figure 10. The dynamic PET images of a female volunteer (#002) after injection of 134 MBq ^{68}Ga-HZ20.

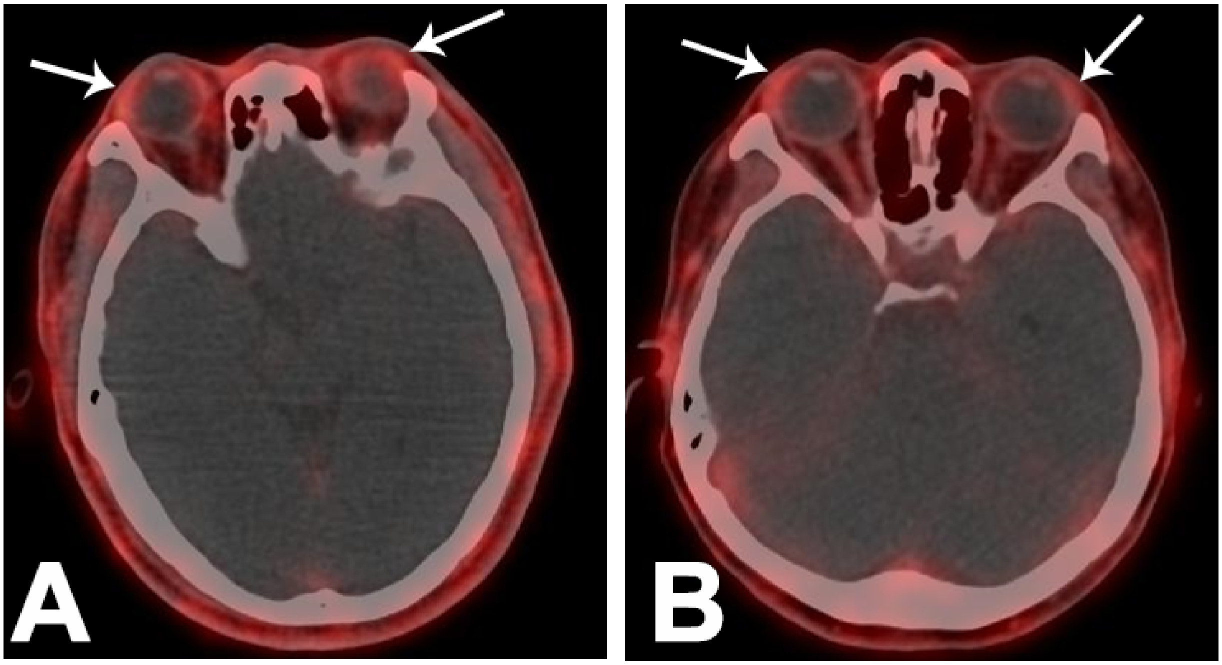

Supplemental Figure 11. Moderate ^{68}Ga-HZ20 uptake in conjunctiva observed in the study. (**A**) A male volunteer (# 009) with SUVmax of 1.5 and (**B**) female volunteer (# 012) with SUVmax of 1.8 are shown as typical examples. The dynamic study showed specific radioactivity uptake in eyes especially conjunctiva although at a medium level. It supports the recent study by Joseph Collin et al. (Collin J, Queen R, Zerti D, et al. *The Ocular Surface*, **2020**, S1542-0124(20)30097-5.), which reported the co-expression of ACE2 and TMPRSS2 was detected in the superficial limbal, corneal and conjunctival epithelium, implicating these as target entry cells for SARS-CoV-2 in the ocular surface. Conjunctiva was indicated by arrows.

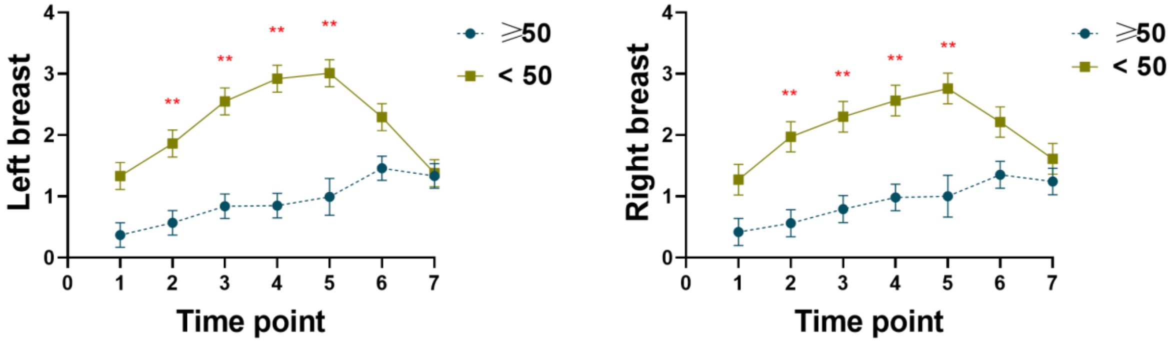

Supplemental Figure 12. Comparison of breast SUVmax between young and old groups at different scanning time. The breast uptake in young female was significantly higher than that of old female at multiple time points (14 min, 23 min, 32 min and 40 min), but this difference tended to dropped at 90 min and 180 min (**: 0.001<P≤0.01).

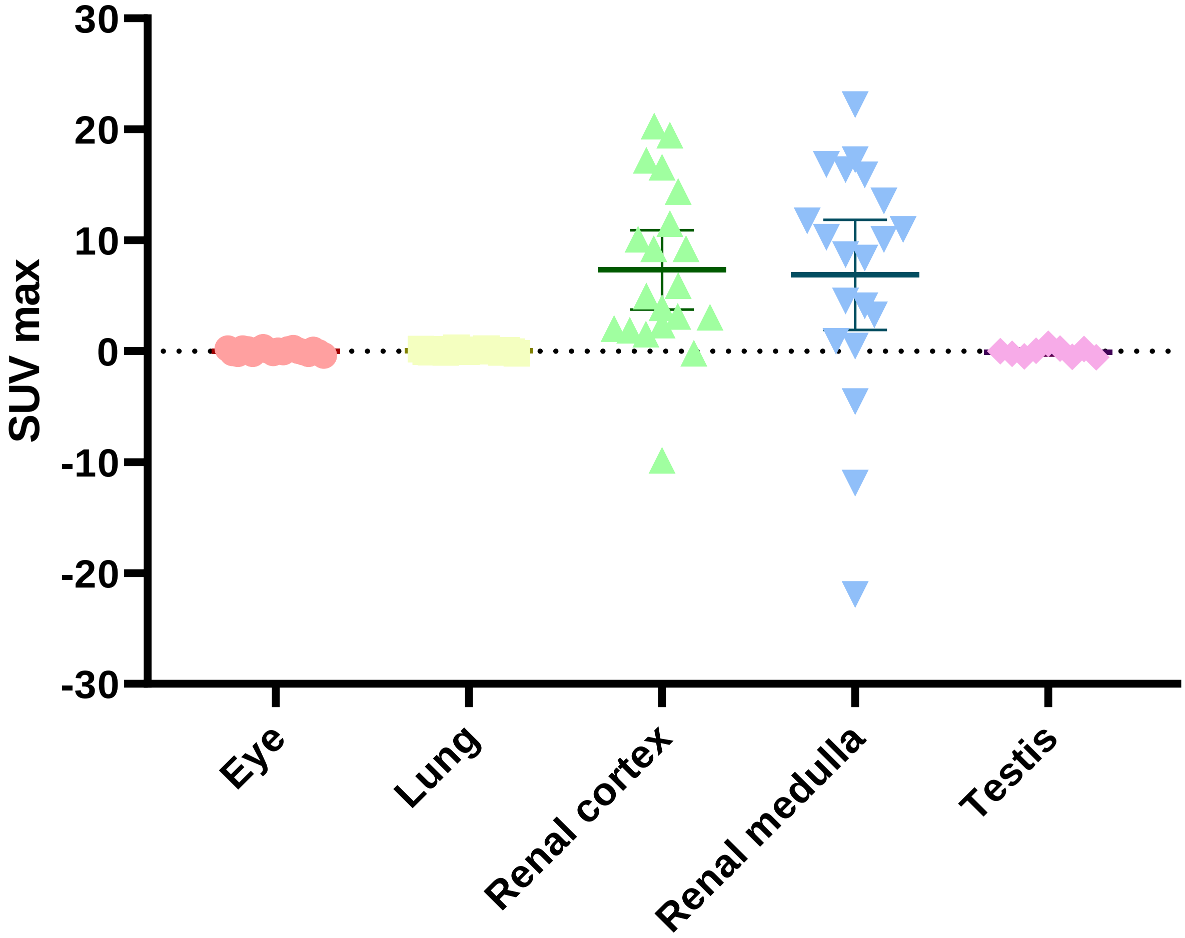

Supplemental Figure 13. Comparison of radioactivity uptake in symmetrical organs. The SUVmax difference between the right and left organ (right minus left) at 90 min imaging. The confidence interval of the eyes (-0.09 to 0.10), lungs (-0.02 to 0.10), and testis (-0.38 to 0.25) which cover zero showed little difference, while renal cortex (3.77 to 10.90) and renal medulla (1.90 to 11.84) which over zero showed an observable higher SUVmax in the right organ.

Table S1. Quality control of ^{68}Ga-HZ20 used in this study.

Parameter	QC Specification	QC Result
**Appearance**	Clear,colorless	pass
**Volume**	2.0-10.0 mL	5.5 mL
**pH**	5.0-8.0	7
**Radio-TLC**	>95%	>99%
**Radio-HPLC**	>95%	>99%
**Ethanol**	<10%	5%
**Endotoxins**	<15 EU/mL	Pass
**Sterility**	Sterile	Pass
**Specific Activity**	40-120 GBq/μmol	60 GBq/μmol

Table S2. General information of volunteers enrolled in this study.

No.	Gender [a]	Weight (kg)	Height (cm)	Age (year)	Injection Dose (MBq)
1	M	73	176	39	155.4
2	F	63	158	32	133.2
3	M	75	173	38	148
4	M	60	162	48	151.7
5	F	61	160	36	170.2
6	F	63	158	65	151.7
7	F	65	158	67	177.6
8	F	65	162	64	185
9	M	75	178	38	192.4
10	F	56	150	72	185
11	M	77	180	38	166.5
12	F	54	155	34	170.2
13	M	72	168	70	185
14	F	58	155	69	122.1
15	F	50	160	34	148
16	M	70	177	63	185
17	F	57	158	62	140.6
18	M	58	168	64	122.1
19	M	65	173	56	177.6
20	F	56	163	34	129.5
21[b]	M	87	178	38	133.2

Note: a) M, Male. F, Female. b) Recovered from the COVID-19.

Movies S1-S3
